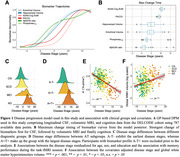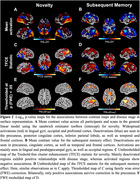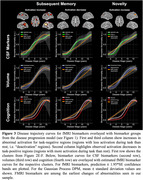# Abnormalities in task‐fMRI activation are related to disease progression towards Alzheimer’s disease

**DOI:** 10.1002/alz.093844

**Published:** 2025-01-09

**Authors:** René Lattmann, Niklas Vockert, Judith Wesenberg, Renat Yakupov, Yanin Suksangkharn, Hartmut Schütze, Matthias Schmid, Peter Dechent, Klaus Fliessbach, Daniel Janowitz, Michael Ewers, Michael T. Heneka, Stefan Hetzer, Oliver Peters, Robert Perneczky, Josef Priller, Klaus Scheffler, Anja Schneider, Annika Spottke, Katharina Buerger, Christoph Laske, Nina Roy‐Kluth, Stefan Teipel, Jens Wiltfang, Steffen Wolfsgruber, Björn H. Schott, Michael Wagner, Frank Jessen, Anne Maass, Gabriel Ziegler, Emrah Düzel

**Affiliations:** ^1^ German Center for Neurodegenerative Diseases (DZNE), Magdeburg Germany; ^2^ Institute of Cognitive Neurology and Dementia Research (IKND), Otto‐von‐Guericke University Magdeburg, Magdeburg Germany; ^3^ German Center for Neurodegenerative Diseases (DZNE), Bonn Germany; ^4^ MR‐Research in Neurosciences, Georg‐August‐University Goettingen, Germany, Goettingen Germany; ^5^ Institute for Stroke and Dementia Research (ISD), University Hospital, LMU, Munich Germany; ^6^ German Centre for Neurodegenerative Diseases (DZNE), Bonn Germany; ^7^ Charité – Universitätsmedizin Berlin, corporate member of Freie Universität Berlin and Humboldt‐Universität zu Berlin, Berlin Germany; ^8^ Charité – Universitätsmedizin Berlin, Berlin Germany; ^9^ LMU University Hospital, Munich Germany; ^10^ Department of Psychiatry and Psychotherapy, Technical University of Munich, Munich Germany; ^11^ University of Tübingen, Tübingen Germany; ^12^ Department of Neurology, University of Bonn, Bonn Germany; ^13^ Institute for Stroke and Dementia Research (ISD), University Hospital, LMU Munich, Munich Germany; ^14^ German Center for Neurodegenerative Diseases (DZNE), Tuebingen Germany; ^15^ Department of Psychosomatic Medicine, University of Rostock, Rostock Germany; ^16^ German Center for Neurodegenerative Diseases (DZNE), Goettingen Germany; ^17^ Department of Neurodegeneration and Geriatric Psychiatry, University Hospital Bonn, Bonn Germany

## Abstract

**Background:**

Differences in task‐fMRI activation have recently been found to be related to neuropathological hallmarks of AD. However, the evolution of fMRI‐based activation throughout AD disease progression and its relationship with other biomarkers remains elusive. Applying a disease progression model (DPM) to a multicentric cohort with up to four annual task‐fMRI visits, we hope to provide a deeper insight into these relationships.

**Method:**

We estimated AD disease stages using a multivariate Gaussian Process (GP) DPM including CSF‐Aß42/40 ratio, CSF‐p‐tau181, hippocampal and entorhinal volume, ADAS13‐Cog sum and PACC5 scores. Disease stages from 493 participants with longitudinal task‐fMRI measurements from DELCODE (165 healthy controls (CN), 214 participants with SCD, 82 with MCI, 32 with suspected AD) were obtained. We derived subsequent memory and novelty contrasts from a visual memory encoding task using general linear modeling (GLM). Contrasts from all available follow‐ups were then submitted to voxel‐based group‐level GLM analyses. Activations from resulting disease‐stage‐related clusters were (1) used to estimate cluster‐level trajectory curves over disease stages using smoothing splines and (2) submitted to linear‐mixed effects models to test longitudinal changes over follow‐ups.

**Result:**

Our DPM‐derived disease stages were associated with clinical groups, fMRI performance and white matter lesions (Figure 1C‐F). Generally, in both contrasts, activation increases were observed in task‐negative clusters while activation decreases were observed in task‐positive clusters (Figure 2C‐F). We did not find indications for inverted u‐shaped associations between disease stage and activation in whole brain voxel‐wise cross‐sectional analyses. However, smoothing splines revealed non‐linear monotonically increasing biomarker abnormality for task‐negative areas, showing earliest changes towards the beginning of disease progression. After a plateau, fMRI activation increases in abnormality conjointly with volume changes. For task‐positive areas, we observed linear relationships with disease stages (Figure 3). Activation changes over follow‐ups were not associated with disease stages.

**Conclusion:**

Biomarker abnormality timing in our DPM reflected hypothetical AD progression. Changes in task‐fMRI activation and deactivation were both associated with progression towards AD. Smoothing spline fits indicated abnormality changes in task‐fMRI activation to begin in the earliest phases of the disease. Findings can be discussed as differential pathophysiological processes such as complex reorganization and neural noise.